# MEMCAIN: a memory-enhanced hybrid CNN-attention model for network anomaly detection

**DOI:** 10.1038/s41598-025-18951-6

**Published:** 2025-10-07

**Authors:** Lan Liu, Tingfeng Cai, Chiyu Zhou, Fengwei Guo, Kundi Yao, Jianhao Zhou

**Affiliations:** 1 Guangdong Polytechnic Normal University, Guangzhou, 510000 China; 2 Department of Electrical and Computer Engineering, University of Waterloo, Waterloo, Canada

**Keywords:** Network anomaly detection, Multi-task learning, Class imbalance, Memory network, Engineering, Mathematics and computing

## Abstract

With increasing cybersecurity threats, effective intrusion detection has become critical for safeguarding networks. Although deep learning methods have advanced, two major issues persist: (1) class imbalance biases models toward normal traffic, increasing false negatives; (2) single-task frameworks limit feature representation and fail to leverage multi-task collaboration potential. To address these, we propose Memory Autoencoder with CNN-Attention Integration Network(MEMCAIN), a multi-task feature fusion deep learning method. First, MEMCAIN integrates CNN with attention mechanisms, constructing CCA Blocks through contrastive normalization to capture spatiotemporal features. These blocks are stacked to form CCANet, enabling comprehensive spatiotemporal feature extraction from traffic data. Second, a memory autoencoder is introduced to capture latent distribution features of traffic flows. Finally, an end-to-end collaborative training framework jointly optimizes CCANet (main task) and the memory autoencoder (auxiliary task). Experiments demonstrate MEMCAIN’s significant superiority over baselines across multiple datasets, with ablation studies validating each module’s efficacy for fine-grained intrusion detection in complex network environments.

## Introduction

According to deployment scenarios and monitoring scope, IDS are divided into host-based HIDS and network-based NIDS. HIDS are deployed on individual terminal devices such as servers and PCs, and monitor local data including system logs and process activities to identify intrusions targeting the device such as malware implantation. However, they have limitations: their monitoring scope is confined to a single host, making them unable to detect cross-device collaborative attacks like DDoS, and they rely on host resources, which leads to high costs in large-scale deployment. By contrast, NIDS are deployed at key network nodes such as gateways and core switches. They achieve global threat perception by analyzing full-network traffic including TCP/IP packets and session interactions. They can not only capture cross-domain attacks beyond the coverage of HIDS, but also provide inter-device communication context data needed for attack chain reconstruction. Moreover, they do not require agent installation on terminals, thus having higher deployment flexibility and adaptability to large-scale networks. Therefore, NIDS is more aligned with the needs of modern network security research. The detection method proposed in this study belongs to the category of NIDS, as it detects threats across the entire network scope by analyzing network traffic.

In recent years, advances in artificial intelligence (AI) have led to the widespread application of machine learning techniques in NIDs, complementing traditional rule-based pattern matching approaches. Existing machine learning-based NIDs employ either traditional machine learning models or deep learning models. However, traditional machine learning models have limitations in handling network traffic data features, making them less suitable for modern NIDs. Deep learning models, on the other hand, can automatically extract high-level feature representations from large-scale network traffic data. As a result, they are more widely adopted in NIDs.

Deep learning-based NIDs can be categorized into supervised and unsupervised learning. Supervised learning employs labeled network traffic data to provide fine-grained detection of known anomalous traffic^1^. Unsupervised learning utilizes unlabeled network traffic data, preserving the diversity of normal network traffic patterns and distinguishing between normal and anomalous traffic to discover unknown network anomalies^2^.

Despite deep learning’s extensive application in NIDs, two primary issues still exist. First, class imbalance in network traffic data is an inherent practical issue: anomalous traffic is typically much less frequent than normal traffic, causing models trained on imbalanced datasets to bias towards the majority class and resulting in higher false-positive rates. Second, existing approaches predominantly employ single-task frameworks, which not only limit the representation power of the extracted features but also fail to leverage the performance enhancement potential from multi-task collaboration, despite evidence suggesting its effectiveness^3,4^.

To address the above issues, this paper proposes Memory Autoencoder with CNN-Attention Integration Network (MEMCAIN), a multi-task feature fusion deep learning method. To overcome the class imbalance problem, we innovatively introduce a memory autoencoder; to tackle the limitations of existing methods in feature representation capability, we innovatively propose CCANet and construct a multi-task learning framework. Specifically, the model integrates the memory autoencoder and CCANet within a multi-task architecture, achieving fine-grained detection of anomalous traffic while discriminating it from normal traffic. The primary and auxiliary tasks mutually enhance performance via end-to-end collaborative learning. The contributions of this work can be summarized as follows:

1. We integrate the attention mechanism with a convolutional neural network (CNN) and introduce contrastive normalization layers, proposing the CCA Block. By stacking multiple CCA Blocks, we achieve efficient spatiotemporal feature extraction from network traffic data. Our approach effectively captures the structural characteristics of network traffic, making it more suitable for large-scale NIDs.
2. We employ CCANet as the main task and the memory autoencoder as the auxiliary task. By leveraging the memory autoencoder’s ability to distinguish normal and abnormal traffic, we fuse the extracted CCANet features with the learned latent traffic features from the memory encoder. This fusion addresses the issue of limited feature representation and alleviates class imbalance.
3. We validate the effectiveness of MEMCAIN through ablation experiments on multiple datasets and compare its performance with several baseline models, demonstrating its superiority in network intrusion detection.

The remainder of this paper is structured as follows: Following this Introduction, the Methodology section systematically introduces the design rationale and key components of the MEMCAIN proposed in this study. Subsequently, the Experiment section details the experimental setup, datasets employed, evaluation metrics, and presents comprehensive experimental results along with in-depth analysis to validate the effectiveness of the proposed approach. Building upon the findings, the Conclusion and future Work section summarizes the main contributions and conclusions of this research, and discusses potential improvements and future research directions. Finally, the paper includes supplementary information in the form of Funding acknowledgements, Data availability, and Contribution.

## Related work

In recent years, intrusion detection systems based on deep learning technology have attracted increasing attention from enterprises and universities. In this section, we will review recent studies on deep learning and multi-task learning in network anomaly detection, the application of memory modules in deep learning, and research on handling imbalanced data.

### Deep learning

Ahmad et al.^5^ compared SVM, RF, and ELM using the NSL-KDD dataset, showing that ELM’s superior classification efficiency makes it more effective for large-scale intrusion detection. Wang et al.^6^ introduced HAST-IDS, which leverages CNNs for spatial feature extraction and LSTMs for temporal modeling, enabling end-to-end feature learning and improving detection accuracy while reducing false positives. Wu et al.^7,8^ proposed LuNet, a spatiotemporal fusion network integrating CNNs and RNNs, which enhances feature extraction and lowers false alarm rates while maintaining high detection accuracy. They also developed Pelican, a deep residual network with skip connections that optimize gradient propagation and eliminate reliance on handcrafted features, demonstrating improved attack detection and reduced false positives. Sinha et al.^9^ combined CNNs and Bi-LSTMs for spatiotemporal modeling, achieving superior performance on NSL-KDD and UNSW-NB15 while significantly lowering false alarms. Gupta et al.^10^ introduced a three-tier intrusion detection framework integrating cost-sensitive deep learning with ensemble learning, using CS-DNN for class imbalance mitigation, XGBoost for anomaly filtering, and RF for fine-grained classification. Cross-dataset evaluations confirm its enhanced recall and high detection accuracy.Vinayakumar et al.^11^ proposed a hyperparameter optimization-based method for selecting DNN topologies, validating its capability in high-dimensional feature abstraction for unknown attack detection. They also developed a dynamic evaluation framework, providing a quantitative basis for assessing the robustness of IDS algorithms in continuously evolving attack scenarios.

Similar to prior spatiotemporal models^6,7,9^, our CCANet adopts CNN and Attention mechanisms for joint spatial-temporal feature learning, but further introduces contrastive normalization layers to adaptively suppress noisy correlations and enhance structural feature discrimination, addressing both class imbalance and traffic complexity in a unified framework.

### Multi-task learning

Multi-task learning (MTL) is a subfield of Deep learning in which multiple learning tasks are solved at the same time, while exploiting commonalities and differences across tasks. This can result in improved learning efficiency and prediction accuracy for the task-specific models, when compared to training the models separately.Inherently, Multi-task learning is a multi-objective optimization problem having trade-offs between different tasks.Lan et al.^12^ addressed class imbalance and the limitations of traditional feature representations by integrating a memory encoder, CNN, and a distance-based prototype network into a multi-task learning model. Extensive experiments on multiple benchmark datasets demonstrated that their proposed model outperforms state-of-the-art baseline methods. Liu et al.^13^introduced a multi-task learning framework that integrates anomaly detection, clustering, and classification. By effectively combining autoencoders with pairwise contrastive learning, they extended the application of autoencoders from unsupervised to supervised learning. Nie et al.^14^ leveraged autoencoders as the shared model architecture in their multi-task learning framework to enhance the performance of the primary task. They also designed a loss-weighting algorithm as the loss function, improving the detection of rare attacks. Telikani et al.^15^ proposed a multi-task learning approach based on a cost-sensitive learning loss strategy. For each task, the hinge loss function was optimized using cost-sensitive learning, effectively leveraging the strengths of different tasks.

Our feature fusion strategy extends^12^’s memory modeling and^13^’s representation learning by bidirectionally aligning CCANet’s structural patterns with memory-induced latent subspaces, achieving both feature enhancement and implicit class balancing.

### Handling imbalanced classes

In network traffic anomaly detection, class imbalance is a common issue where normal traffic samples significantly outnumber anomalous attack samples. This imbalance negatively impacts the classification performance of models, making it difficult to identify anomalous traffic instances. There are two main approaches to addressing this issue. The first involves data preprocessing, focusing on balancing the minority class. The second approach involves modifying the algorithm or model itself, emphasizing the optimization of the model’s learning capability for the minority class. Specifically, this approach does not directly alter the data distribution but improves the model’s training strategy or introduces specific mechanisms to enhance its sensitivity to anomalous samples. This paper adopts the second approach. Abdulrahman et al.^16^ designed a CNN-based intrusion detection system for Industrial IoT environments and employed Synthetic Minority Over-sampling Technique (SMOTE) to balance the dataset. Andresini et al.^17^ proposed a GAN- and CNN-based network traffic anomaly detection method, using GANs to augment imbalanced network traffic data, thereby training a more robust anomaly detection model. Ding et al.^18^ proposed a data augmentation method using a GAN with a multi-generator structure, capable of generating various types of attack data to train models and improve performance. Ren et al.^19^ proposed a CNN-based neural network with an attention mechanism and introduced the Equalization Loss function (EQLv2). By dynamically adjusting the weights of minority samples, their approach mitigated the adverse impact of class imbalance. Experimental results demonstrated that their method reduced false alarm rates and improved detection accuracy.

While data augmentation techniques like SMOTE and GANs offer a direct way to address imbalance, they present significant challenges in the network security domain:

1. SMOTE limitations: SMOTE and its variants generate synthetic samples by interpolating between existing minority instances. This can lead to the creation of unrealistic or noisy samples that do not accurately reflect the true, often complex and non-linear, distribution of real attack traffic, potentially degrading model performance or introducing generalization issues.
2. GAN limitations: Models trained predominantly on GAN-generated data risk learning spurious patterns inherent to the generation process rather than true attack signatures. This synthetic overfitting degrades performance when detecting real-world attacks, potentially increasing false negatives.

In contrast to existing methods that relied on data augmentation or loss reweighting for class balance, our approach proposed the CCA Block, integrating attention mechanisms with contrastive normalization to adaptively capture structural correlations in network traffic and inherently mitigate imbalance through feature-space regularization. Essentially resolving the “Synthetic Sample Dependency Syndrome” that plagues generalization performance.

## Methodology

In this section, we introduce the design of MEMCAIN framework.

### The structure of MEMCAIN

**Fig. 1:**
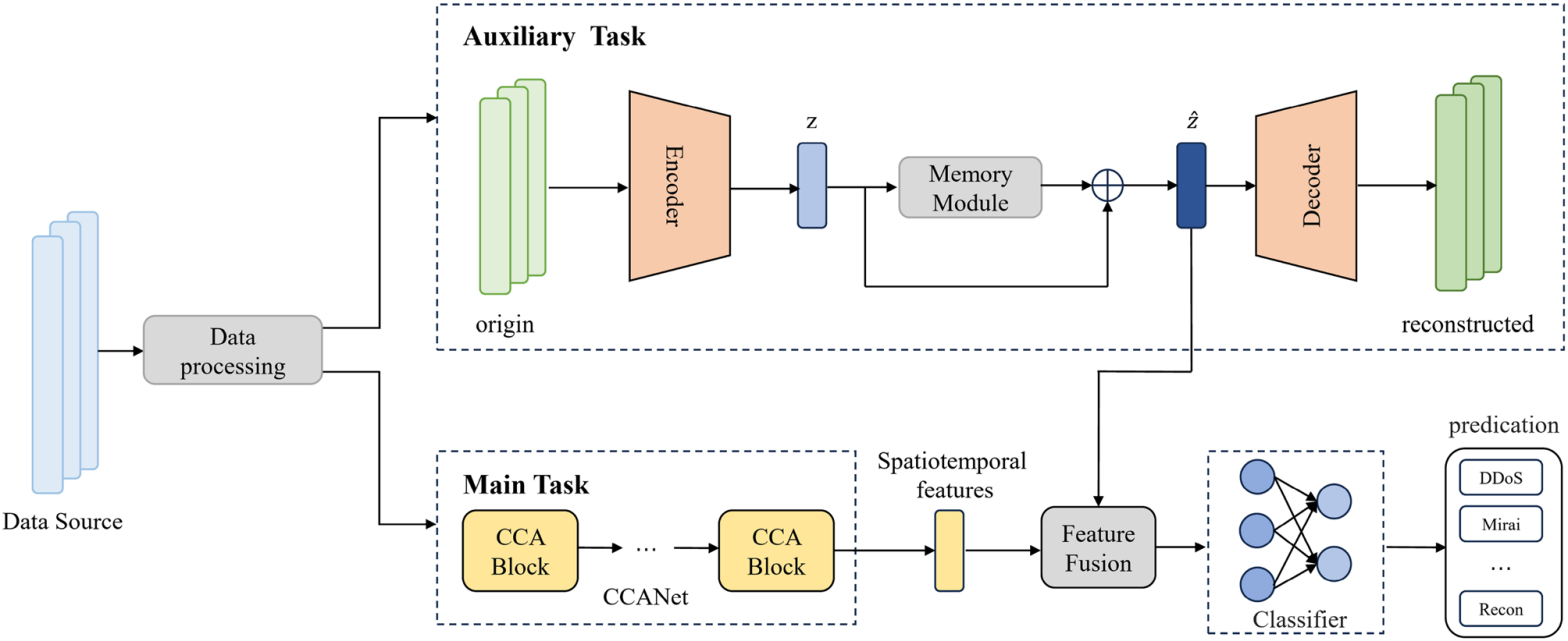
The structure of MEMCAIN.

The structure of our proposed multi-task learning network architecture (MEMCAIN) is illustrated in Fig. 1. MEMCAIN consists of three main components: **data preprocessing**, a memory-augmented autoencoder (MEMAE) as the **auxiliary task**, and CCANet as the **main** **task**. The workflow of MEMCAIN proceeds as follows:

First, in the data preprocessing component, the raw data is preprocessed and sampled to enhance its quality. Subsequently, the preprocessed data is fed in parallel to the two task components:

1. For the auxiliary task, we construct an end-to-end unsupervised learning network based on memory autoencoders to learn latent traffic features from the data.
2. For the main task, we develop CCANet, composed of CCA Blocks, to extract spatiotemporal features from the data.

Following this, a feature fusion module integrates the latent traffic features learned by the auxiliary task with the spatiotemporal features extracted by the main task. Therefore, we apply the dilated self-attention mechanism to process the concatenated feature vector, which dynamically adjusts receptive fields to capture both local details and global dependencies. This generates refined embedding tokens with enhanced discriminative power.

Finally, these embedding tokens are utilized by a classifier to perform anomaly detection.

### Data preprocessing

Before training and testing the model, we preprocess the source to eliminate outliers that may interfere with the model, thereby accelerating convergence and improving accuracy. Unlike data augmentation methods^16–18^ , our preprocessing does not require sampling to address data imbalance. Instead, we leverage multi-task learning to mitigate data imbalance, reducing preprocessing time. Our data preprocessing consists of the following three steps:

1. Data cleaning: The dataset may contain ‘NAN’, ‘INF’, or missing values, which cannot be directly fed into the model as they may cause training errors. To address this, we replace all outliers and missing values with zero.
2. One-hot encoding: The dataset may contain textual symbols such as “https” and “http”. One-hot encoding converts categorical variables into mutually exclusive binary vectors, preventing misleading relationships between categories.
3. Normalization: Normalization prevents gradient instability and computational errors caused by large feature value ranges. Therefore, we apply min-max normalization to scale feature values to the [0,1] range. The formula for min-max normalization is as follows:
  1 $$\begin{aligned} X_{\textrm{norm}} = \frac{X - X_{\min }}{X_{\max } - X_{\min }} \end{aligned}$$
  where $X$,$X_{min}$,$X_{max}$ represent the original data value, Minimum value in the dataset and Maximum value in the dataset.

The final dimensionality of the CICIDS2017 dataset data input to the model is 77, and that of the KDD dataset data is 122.

### Multi-task learning

#### MEMAE

MEMAE is an unsupervised learning model. Serving as an auxiliary task, this architecture leverages the structural characteristics of its specialized compression–reconstruction mechanism and the memory module’s addressing capabilities to amplify core distinctions between traffic categories, thereby making latent features of different traffic types highly distinct. The extracted latent features are integrated into the main task’s feature space, addressing the inherent limitations of feature representation capability in existing methods.

**Fig. 2:**
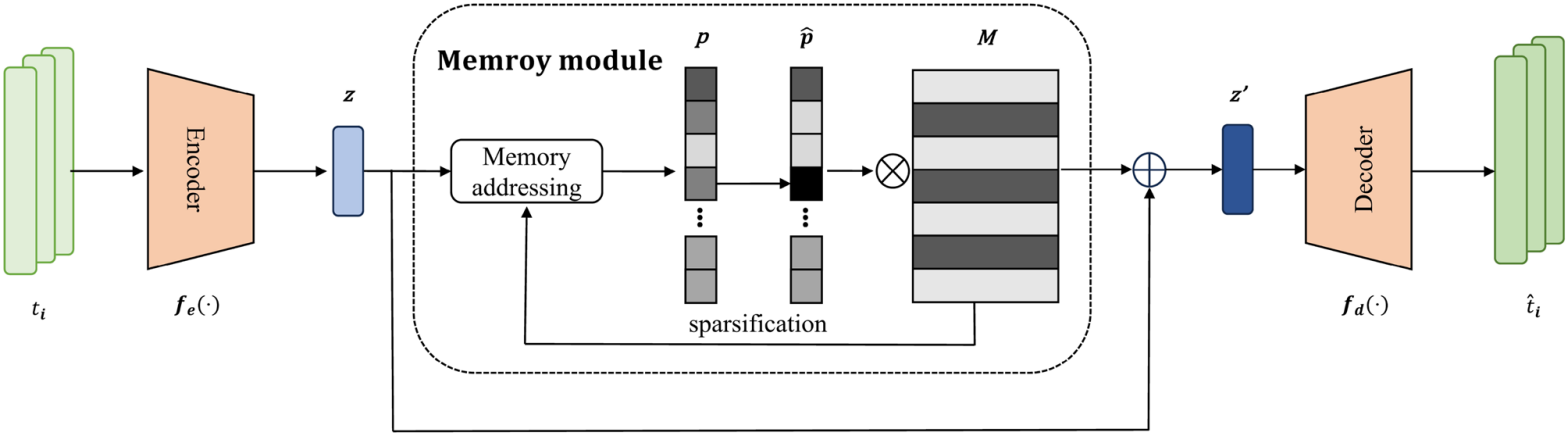
The architecture of MEMAE. It primarily consists of three components: an encoder, a memory module, and a decoder.

As shown in Fig. 2, MEMAE consists of three main components: an encoder, a memory module, and a decoder. The encoder compresses the input data into a low-dimensional latent space.

The memory module stores and retrieves latent traffic patterns. The decoder reconstructs the data. The encoder and decoder form a symmetric structure to reconstruct latent traffic features to match the original traffic data size for loss calculation. The memory module employs an attention-based similarity computation mechanism to retrieve the most relevant memory entries. The selected memory entries are concatenated with the encoder-generated latent representations and subsequently passed to the decoder to aid reconstruction.

We define the overall sample feature space as $T$ and the latent encoded sample feature space as $Z$ . For each sample feature $t_{i} \in T$ , the encoder compresses it into a latent sample feature $z_{i} \in Z$. For each latent sample feature $z' \in Z$, the decoder reconstructs it into $\hat{t}_{i} \in T$.

2 $$\begin{aligned} z&= f_{\textrm{encoder}}(t_{i}, \theta _{e}) \end{aligned}$$

3 $$\begin{aligned} \hat{t}_{i}&= f_{\textrm{decoder}}(z', \theta _{d}) \end{aligned}$$

where $\theta _{e}$ and $\theta _{d}$ are the trainable parameters of the encoder and decoder, respectively.

The memory module enhances the encoder’s ability to model complex data, enabling rapid recall of key features.

The memory module is defined as a random matrix $M \in \mathbb {R}^{N \times S}$, where *N* the number of memory slots denotes the total number of independent memory entries corresponding to memory size in implementation and *S* the slot width represents the feature dimension of each memory entry equivalent to the dimension of memory values. Each memory entry $m_j \in [1, N]$ is retrieved from the *j*-th row of *M*. The module employs separate keys and values with memor keys of shape $(N, \text {key\_dim})$ computing attention weights and memory values of shape (*N*, *S*) providing actual memory content. For reading, the latent sample feature interacts with memory keys via dot product to generate attention weights which are then used to weight-sum memory values to produce the memory-enhanced feature $z'$. Specifically, the latent sample feature $z'$ enhanced by the memory module can be expressed as follows :

4 $$\begin{aligned} z' = z + wM = z + \sum _{j=1}^{N} w_j m_j \end{aligned}$$

where $w \in \mathbb {R}^N$ represents the attention weight vector, and $w_j$ denotes the *j*-*th* element of *w*, computed using cosine similarity between *z* and $m_j$:

5 $$\begin{aligned} w_j = \frac{\exp \left( c(\textbf{z}, \textbf{m}_j)\right) }{\sum _{q=1}^N \exp \left( c(\textbf{z}, \textbf{m}_q)\right) } \end{aligned}$$

where ${C(\cdot )}$ represents cosine similarity.

6 $$\begin{aligned} c(\textbf{z}, \textbf{m}_j) = \frac{\textbf{z} \textbf{m}_j^\top }{\Vert \textbf{z}\Vert \Vert \textbf{m}_j\Vert } \end{aligned}$$

Additionally, the attention weight matrix *w* needs to be sparsified to ensure the encoder focuses on key features during reconstruction, preventing interference from irrelevant combinations. Similar to Gong et al.^20^, we employ a hard thresholding approach for sparsifying the attention weight matrix, but we introduce a weight difference term $(w_{i} - \tau )$ to enhance the strictness of sparsification.

$$\begin{aligned} \hat{w}_{i} = \frac{ \max (w_{i} - \lambda , 0) \cdot (w_{i} - \tau ) }{ \left| w_{i} - \lambda \right| + \epsilon } \end{aligned}$$

where $\epsilon$ represents a small positive scalar, $\epsilon$ is a threshold chosen from the range [1/*N*, 3/*N*], and $\max (\cdot )$ corresponds to the ReLU activation function. After sparsification, $\hat{w}$ is re-normalized as follows:

7 $$\begin{aligned} \hat{w}_i&= \frac{\hat{w}_i}{\left\| \hat{\varvec{w}} \right\| _1} \end{aligned}$$

Minimizing the entropy of attention weights sharpens their distribution. We employ a negative entropy-based loss term as the sparsification loss function:

8 $$\begin{aligned} L_{\text {spar}}&= -\sum _{i=1}^{N} \hat{w}_i \log \left( \hat{w}_i \right) \end{aligned}$$

To enable MEMAE to learn more representative features from the data, we use mean squared error (MSE) as the reconstruction loss:

9 $$\begin{aligned} L_{\textrm{MSE}} = \frac{1}{n} \sum _{i=1}^n \left( \hat{t}_i - t_i \right) ^2 \end{aligned}$$

The total MSE loss is obtained from Eq. (8) and Eq. (9) as follows:

10 $$\begin{aligned} L_a = \delta L_{\textrm{spar}} + L_{\textrm{mse}} \end{aligned}$$

where $\delta$ denote a hyperparameter. In this work, we set it to 0.01 for training MEMAE.

#### CCANet

**Fig. 3:**
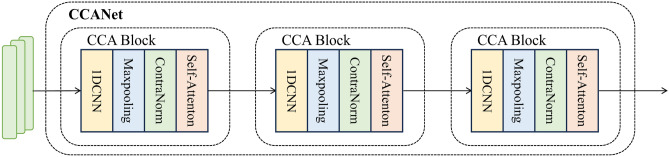
The overall structure of CCANet, which consists of three CCA Blocks. Each CCA Block consists of a 1D-CNN, a Max-pooling layer, a Contra Norm layer, and an Attention mechanism.

Existing approaches to address class imbalance typically employ data augmentation techniques such as GANs. However, models trained on GAN-generated data may learn spurious patterns inherent in the generation process rather than authentic attack characteristics, which degrades performance in detecting real attacks and potentially increases the false negative rate. To resolve this issue, our research introduces innovations at the model level. While current models for handling class imbalance primarily utilize CNNs and LSTMs to leverage spatial and sequential features for modeling, and their limited ability to capture long-range dependencies in ultra-long sequences remain critical bottlenecks. Therefore, we propose CCANet, a novel deep learning architecture tailored for spatiotemporal feature extraction in network traffic analysis.

Specifically, we propose the CCA (CNN-ContraNorm-Attention) Block, a triple-enhanced feature extraction module. As shown in Fig. 3 , the block cascades a 1D convolutional layer, a contrastive normalization (ContraNorm) layer, and an attention mechanism into a cohesive pipeline.

Figure 3 shows that the first two layers of the CCA Block consist of a 1D-CNN and a Max-pooling layer. The 1D-CNN extracts spatial features from input samples, while Max-pooling reduces the dimensionality of the feature map and the number of parameters in subsequent layers, thereby mitigating the risk of overfitting. The operations of CNN and Max-pooling on data are formally described in Eqs. (11) and (12).

11 $$\begin{aligned} x_{n} = f_{\textrm{relu}} \left( f_{\textrm{conv}} \left( W_{n} \cdot t_{n} + b_{n} \right) \right) \end{aligned}$$

12 $$\begin{aligned} y_{i} = \max (x_{i}) \end{aligned}$$

where $t_{n}$ represents the input features of the *n*-*th* CCA Block, and when *n*=1, it corresponds to the raw data. $x_n$ denotes the output of the *n*-*th* CCA Block. $w_n$ and $b_n$ represent the weight matrix and bias parameters, respectively.

To enhance training stability and mitigate overfitting, we apply contrast normalization to adjust the outputs of 1D-CNN and Max-pooling:

13 $$\begin{aligned} y_n = \frac{y_i}{\Vert y \Vert _2} \end{aligned}$$

14 $$\begin{aligned} \textrm{S}= \textrm{ContraNorm}(y) = \textrm{LayerNorm}\left( y - \gamma \left( \textrm{Softmax}\left( \frac{y_n y_n^\top }{\tau } \right) y \right) \right) \end{aligned}$$

where $y_n$ represents the normalized feature of the *n*-*th* CCA Block, calculated using the $\ell _2$-norm. The term $\frac{y_n y_n^\top }{\tau }$ is used to calculate the similarity weights between features. $\textrm{softmax}(\cdot )y$ computes the weighted attention matrix. The function $\textrm{LayerNorm}(\cdot )$ denotes layer normalization, which helps maintain training stability. $y - \gamma (\cdot )$ introduces residual subtraction for correction, enhancing feature discriminability. $\gamma$ and $\tau$ are hyper parameters, set to 0.1 and 1 in practice, yielding promising results. In the final layer of the CCA Block, we incorporate a self-attention mechanism for connection.

15 $$\begin{aligned} y' = \textrm{Attention}(Q, K, V) = \textrm{softmax}\left( \frac{QK^\top }{\sqrt{d_{k}}} \right) V \end{aligned}$$

where *Q*, *K*, *V* represent the query, key, and value matrices, respectively, respectively, which are generated through independent linear transformations.

16 $$\begin{aligned} Q = S \cdot W_Q + b_Q \end{aligned}$$

17 $$\begin{aligned} K = S \cdot W_K + b_K \end{aligned}$$

18 $$\begin{aligned} V = S \cdot W_V + b_V \end{aligned}$$

where$S \in \mathbb {R}^{B \times L \times d_{\text {in}}}$ denotes the output of contrast normalization, with *B* as batch size, *L* as input sequence length, and $d_{\text {in}}$ as input dimension; $W_Q, W_K, W_V \in \mathbb {R}^{d_{\text {in}} \times d_{\text {k}}}$ are learnable weight matrices for the linear transformations of *Q*, *K*, and *V*, respectively; $b_Q, b_K, b_V \in \mathbb {R}^{d_{\text {k}}}$ are trainable bias terms corresponding to $W_Q$, $W_K$, and $W_V$; $d_k$ represents the dimensionality of the input features, while $\frac{1}{\sqrt{d_k}}$ serves as the scaling factor.

After applying the self-attention mechanism, the CCA Block captures more comprehensive global contextual information.

#### Feature fusion and classification

**Fig. 4:**
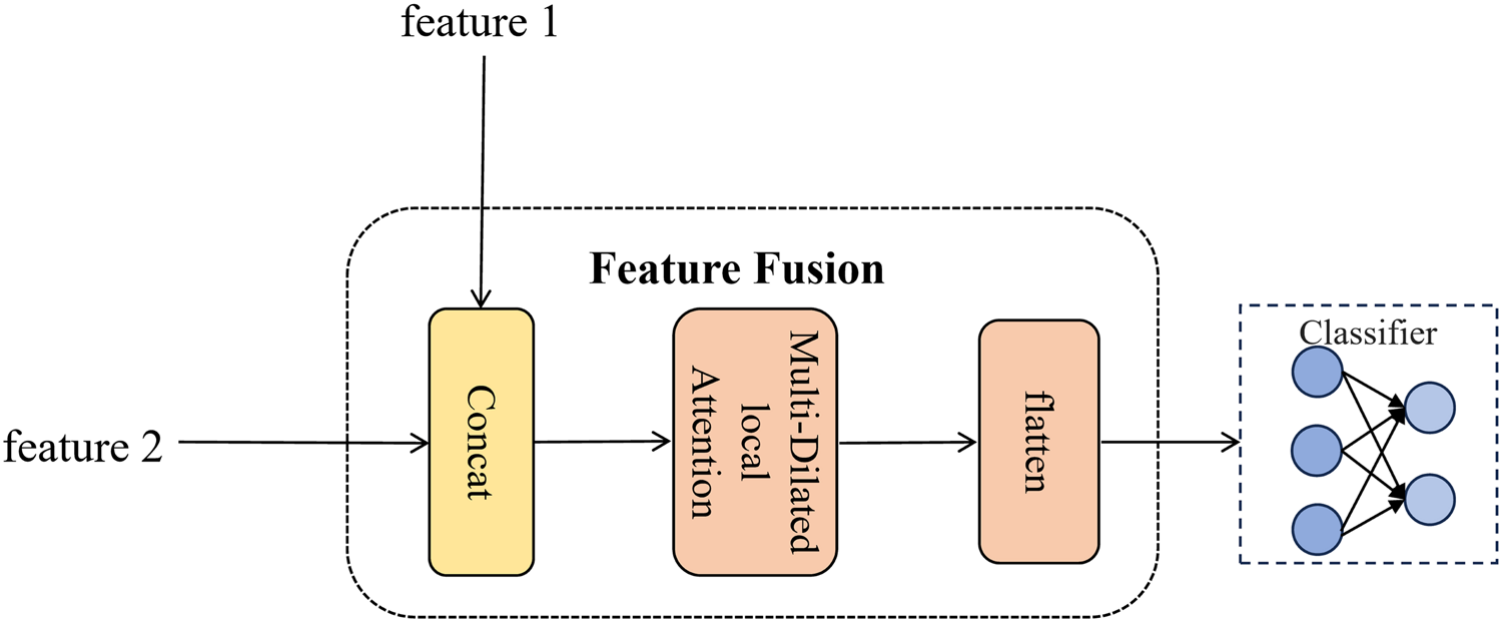
The feature fusion block, which consists of three components: Concat, Multi-Dilated local Attention, and flatten.

In the proposed multi-task learning framework, the feature fusion block is responsible for obtaining the final feature vector. Specifically, the compressed feature representation from MEMAE is denoted as $z'$, while the spatiotemporal features extracted by CCANet are represented as $y'$. The final feature vector obtained through multi-task learning is expressed as (Fig. 4):

19 $$\begin{aligned} X = \textrm{Concat}(z', y') \end{aligned}$$

Therefore, we apply the Multi-Dilated local attention mechanism to process the concatenated feature vector *X*. The dilated self-attention mechanism is formulated as follows:

20 $$\begin{aligned} X_{\text {window}}^{(h,d)}[i] = \left\{ X\left[ i + d \cdot m \right] \;\Bigg |\; m \in \left\lfloor -\frac{k-1}{2}, \frac{k-1}{2} \right\rfloor \right\} \end{aligned}$$

21 $$\begin{aligned} Q_h^{(d)} = X_{\text {window}}^{(h,d)} W_q^h, \quad K_h^{(d)} = X_{\text {window}}^{(h,d)} W_k^h, \quad V_h^{(d)} = X_{\text {window}}^{(h,d)} W_v^h \end{aligned}$$

22 $$\begin{aligned} X_o = \textrm{MultiDilatedLocalAttention}(X) = \textrm{Proj} \Biggl ( \frac{1}{k} \sum _{m=1}^k \biggl [ \textrm{Concat} \biggl ( \biggl \{ \textrm{Softmax} \biggl ( \frac{Q_h^{(d)}{K_h^{(d)}}^{\!\top }}{\sqrt{C/H}} \biggr ) V_h^{(d)} \biggr \}_{h=1}^H \biggr ) \biggr ] \Biggr ) \end{aligned}$$

where *h* denotes the index of the attention head, *H* is the total number of attention heads, *d* represents the dilation rate, *k* is the local window size, and *C*/*H* corresponds to the feature dimension per head. $X_{\text {window}}^{(h,d)}[i]$ represents the extracted dilated window, where each attention head independently learns $W_q^h$, $W_k^h$, $W_v^h$. $\textrm{Concat}(\cdot )_{h=1}^{H}$ denotes the aggregation and concatenation of the *H* attention heads. $\textrm{Proj}(\cdot )$ represents the projection layer.

Subsequently, a fully connected layer with $\textrm{softmax}$ as the activation function is used as the classifier to produce the final classification output $\hat{y}$.

23 $$\begin{aligned} \hat{y}=argmax(softmax(W_{c}X_{o}+b_{c})) \end{aligned}$$

where $W_c$ and $b_c$ denote the trainable parameters of the fully connected layer.

In the main task training, we use cross-entropy as the loss function:

24 $$\begin{aligned} L_{b}=-\frac{1}{N} \sum _{n=1}^N \sum _{i=1}^C y_{n,i} \log (\hat{y}_{n,i}) \end{aligned}$$

where $y_{n,i}$ and $\hat{y}_{n,i}$ represent the one-hot encoded ground-truth labels and the predicted probability distribution, respectively. $N$ and $C$ denote the batch size and the number of classes, respectively.

Finally, based on Eqs. (10) and (24) the total loss of MEMCAIN is defined as:

25 $$\begin{aligned} L=\alpha L_a+\beta L_b \end{aligned}$$

$\alpha$ and $\beta$ serve as hyperparameters. Empirically, setting $\alpha$ to 0.9 and $\beta$ to 1.2 produced optimal performance.

### Comparative analysis with existing methods

To demonstrate the superiority of our approach, we conduct comparisons with state-of-the-art deep learning methods.

LuNet^7^ adopts a hierarchical architecture that integrates CNN and RNN, where each hierarchical level contains a combined CNN-RNN module to enable synchronous learning and collaborative capture of spatial and temporal features. Batch normalization is applied between the CNN output and RNN input within each LuNet Block to accelerate training convergence and improve accuracy. Compared to LuNet, while drawing inspiration from its idea of combining different foundational neural networks, our proposed model employs a CNN-Attention hybrid structure in its primary task, which demonstrates enhanced effectiveness in extracting spatiotemporal features from traffic data relative to LuNet.

Pelican^8^ proposes a deep residual network based on CNN and GRU, pioneering the application of residual learning in network anomaly traffic detection to mitigate performance degradation during training of its designed deep neural network, enabling deeper networks to maintain or even improve performance. Compared to Pelican, we incorporate its concept of residual connections in the network traffic anomaly detection domain by introducing residual connections to the Memory Autoencoder in the auxiliary task of our model, achieving efficient extraction of latent features in traffic data.

M2VT-IDS^14^ designs a multi-view shared network with task-specific attention mechanisms. Its multi-view shared network combines LSTM and Transformer to extract generic high-dimensional features for cross-task feature sharing, while task-specific attention networks implement simultaneous multi-task training via distinct attention modules for three subtasks. In contrast to M2VT-IDS’s parameter-hard-sharing-based multi-task learning framework, our proposed model adopts a soft-sharing-based multi-task framework, enhancing overall performance through collaborative training of primary and auxiliary tasks.

MEMBER^12^ proposes a multi-task learning framework comprising a CNN-based primary task, a prototype network DPNet-based auxiliary task, and a memory autoencoder-based auxiliary task. Compared to MEMBER, while leveraging its multi-task learning framework and MEMAE architecture, we refine the encoder and decoder components of the memory autoencoder. Specifically, we design a primary task centered on CCANet and an auxiliary task grounded in MEMAE, which thoroughly exploits contextual dependencies among network traffic and elevates detection effectiveness.

## Experiment

**Fig. 5:**
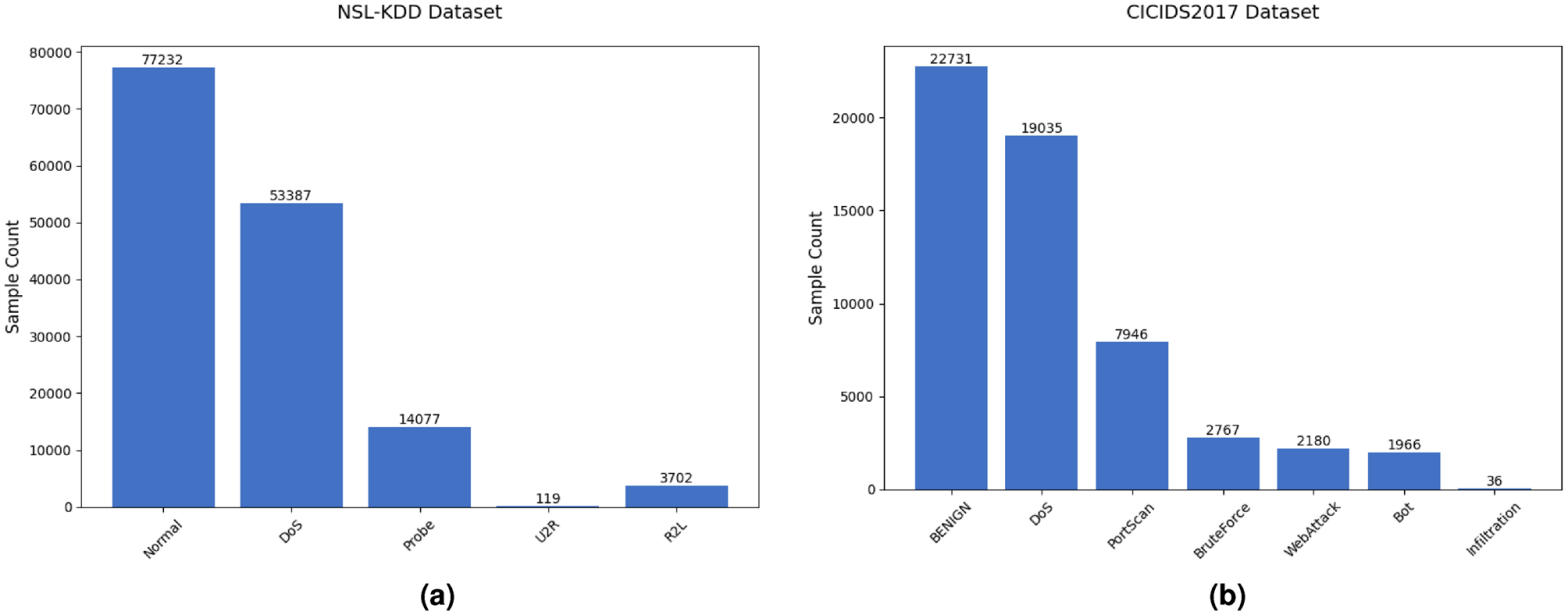
Class distribution.

In this section, we evaluate the proposed model on the NSL-KDD and CICIDS2017 datasets. The sample distributions of the two datasets are shown in Fig. 5. The dataset descriptions are as follows:

The NSL-KDD dataset is an improved version of the KDD99 dataset, addressing inherent issues such as redundant records and class imbalance. It is extensively utilized for benchmarking network intrusion detection systems and contains labeled network traffic divided into normal and attack categories. The dataset comprises 41 features, including numerical and categorical attributes. Each record is classified into one of five major categories: Normal, DoS, Probe, R2L, and U2R.

The CICIDS2017 dataset is an intrusion detection benchmark dataset designed to simulate real-world network environments. It includes normal traffic and various emerging attack types such as BruteForce, DoS, Heartbleed, Web attacks, and DDoS. Each traffic record is labeled based on timestamp, IP address, port, protocol, and attack type. The dataset was collected over five business days from July 3 to July 7, 2017. The first day consists solely of normal traffic, whereas the subsequent four days feature scheduled injections of eight attack types, including penetration attacks and botnets. Since DDoS and DoS attacks share the primary objective of exhausting target system resources to disrupt services, they exhibit high similarity in attack behavior and impact. Therefore, in this experiment, they are merged into the DoS category.

We use accuracy, precision, recall, false positive rate and F1-score as performance metrics, which are defined as follows:

26 $$\begin{aligned} ACC=\frac{TP+TN}{TP+TN+FP+FN} \end{aligned}$$

$ACC$ is the proportion of correctly predicted samples to the total number of samples.

27 $$\begin{aligned} PRE=\frac{TP}{TP+FP} \end{aligned}$$

$PRE$ is the proportion of true positive instances among the samples predicted as positive.

28 $$\begin{aligned} REC=\frac{TP}{TP+FN} \end{aligned}$$

$REC$ is the proportion of correctly predicted positive instances to the actual positive instances.

29 $$\begin{aligned} FPR=\frac{FP}{FP+TN} \end{aligned}$$

$FPR$ is the proportion of negative instances incorrectly predicted as positive among all actual negative instances.

30 $$\begin{aligned} F1=\frac{2TP}{2TP+FP+FN} \end{aligned}$$

$F1$ is the harmonic mean of Precision (Pre) and Recall (Rec).

$TP$, $TN$, $FP$, and $FN$ represent the four fundamental metrics: true positive, true negative, false positive, and false negative, respectively.

In our proposed MEMCAIN model, we set the number of neurons in the first two fully connected layers of the MEMAE encoder to 128 and 16, respectively, with a memory size of 128. Additionally, in CCANet, the kernel sizes of the CNN layers in the three CCA Blocks are set to 32, 64, and 128, respectively, while the convolution kernel size is uniformly set to 32. Each kernel has a stride of 1, and the padding is set to “SAME”. For the feature fusion module, we configure the dilated self-attention heads to 8, the local window size to 3, and the dilation rate to 1.

The training parameter settings of the model are presented in Table 1. All experiments in this study are conducted on a small-scale server equipped with an Intel i5-12400 CPU, an 8GB NVIDIA GeForce RTX 4060 GPU, and 32 GB of RAM.

**Table 1:** Settings of model training parameters.

Parameter	Details
Batch size	128
Epochs	100
Optimizer	Adam
Dataset split ratio	Training:Validation:Test = 8:1:1
Random seed	42
EarlyStopping	Monitors val_output_loss; stops after 10 epochs without improvement (min_delta=0.0001); mode=‘min’;
Learning rate strategy	Initial learning rate of 0.001 (first 20 epochs); from the 21st epoch onward, exponential decay with a base of $e^{-1}$

### Model results

We evaluated the model’s performance through 10 rounds of multi-class classification experiments, where multi-class classification refers to the model’s task of determining whether a sample belongs to a specific attack category or the normal category within the dataset.

The results of 10 rounds of multi-class classification experiments on the CICIDS2017 and KDD datasets are presented in Table 2. To evaluate the model’s robustness, we compare the standard deviations (Std Dev) of metrics across datasets: NSL-KDD exhibits lower Std Dev for all indicators—ACC (0.021 vs. 0.031 for CICIDS2017), REC (0.069 vs. 0.791), and FPR (0.008 vs. 0.011). The drastic difference in REC’s Std Dev underscores the model’s more stable performance on NSL-KDD, likely due to its simpler sample distribution compared to CICIDS2017’s diverse, complex traffic patterns.

**Table 2:** Statistical evaluation of model performance across 10 runs on NSL-KDD and CICIDS2017 test datasets.

Metric	CICIDS2017		NSL-KDD	
	Mean (%)	Std Dev (%)	Mean (%)	Std Dev (%)
ACC	99.08	0.031	99.48	0.021
REC	97.07	0.791	93.57	0.069
FPR	0.17	0.011	0.17	0.008

**Fig. 6:**
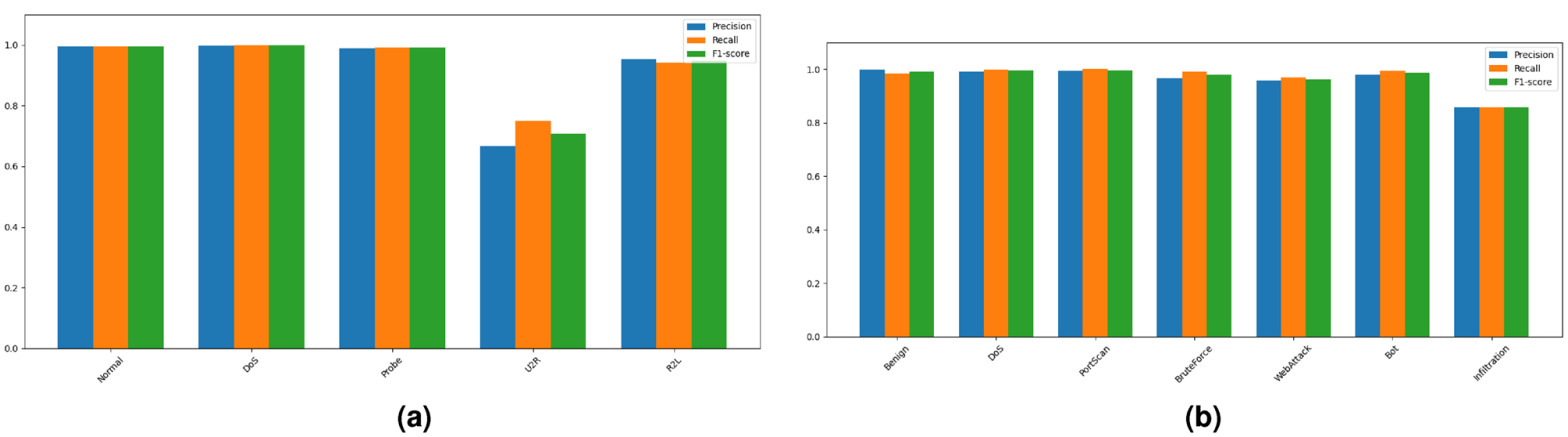
Category metrics.

The multi-class classification results are presented in Tables 6 and 7.On the NSL-KDD dataset, the ACC is 99.48, REC is 93.57, and FPR is 0.17. For the CICIDS2017 dataset, the ACC is 99.08, REC is 97.07, and FPR is 0.17. Figure 6a,b illustrate the PRE, REC, and F1 metrics for each category on NSL-KDD and CICIDS2017 datasets respectively.

**Fig. 7:**
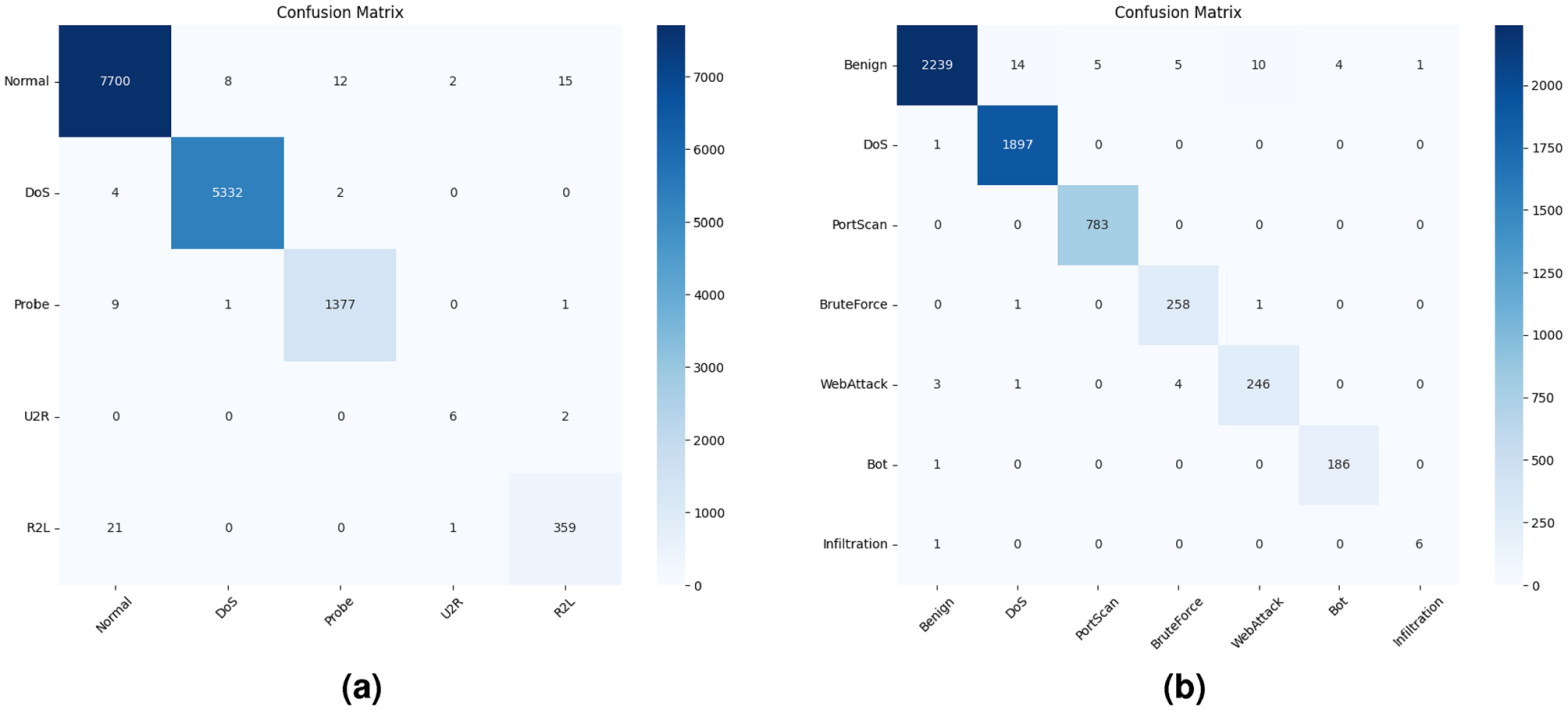
Confusion matrix.

**Fig. 8:**
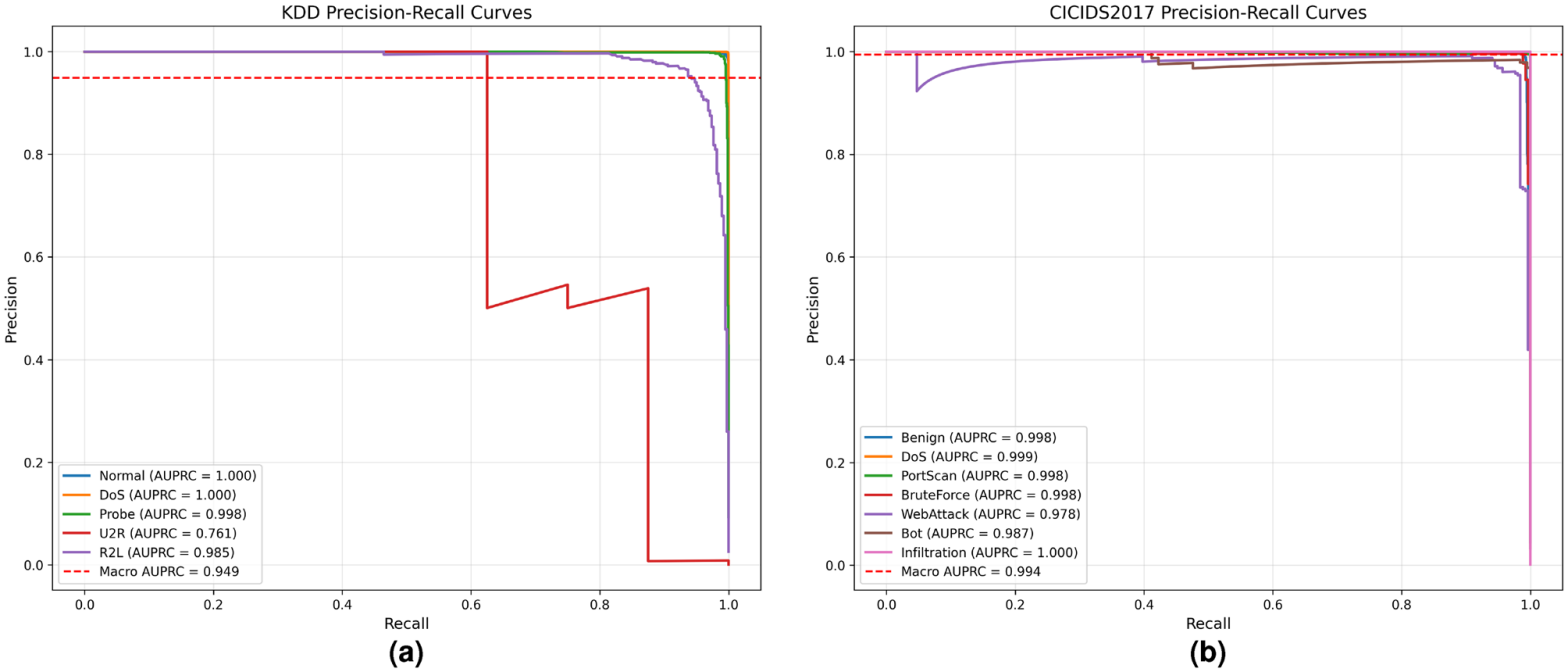
Precision-recall curves.

As shown in the figures, our proposed method exhibits excellent performance in malicious traffic detection. To better visualize the model results, we present the confusion matrix to shows the multi-class classification results on the NSL-KDD dataset, where most test samples are concentrated along the diagonal, indicating a high overall accuracy.

For the NSL-KDD dataset, as shown in the diagonal of the confusion matrix in Fig. 7a, our model successfully detects Normal traffic, as well as DoS, Probe, U2R, and R2L traffic.

Notably, although the number of U2R attack samples is relatively small compared to other categories, the model still achieves a sufficient recall rate, as indicated in Fig. 6a . This observation suggests that our model is capable of mitigating the class imbalance issue. Similarly, based on the experimental results on CICIDS2017 as presented in Figs. 6b and 7b, we observe that the model can effectively detect malicious traffic even only a few infiltration attack samples is available.

For the NSL-KDD dataset, as shown in the diagonal of the confusion matrix in Fig. 7a, our model successfully detects Normal traffic, as well as DoS, Probe, U2R, and R2L traffic.

**Fig. 9:**
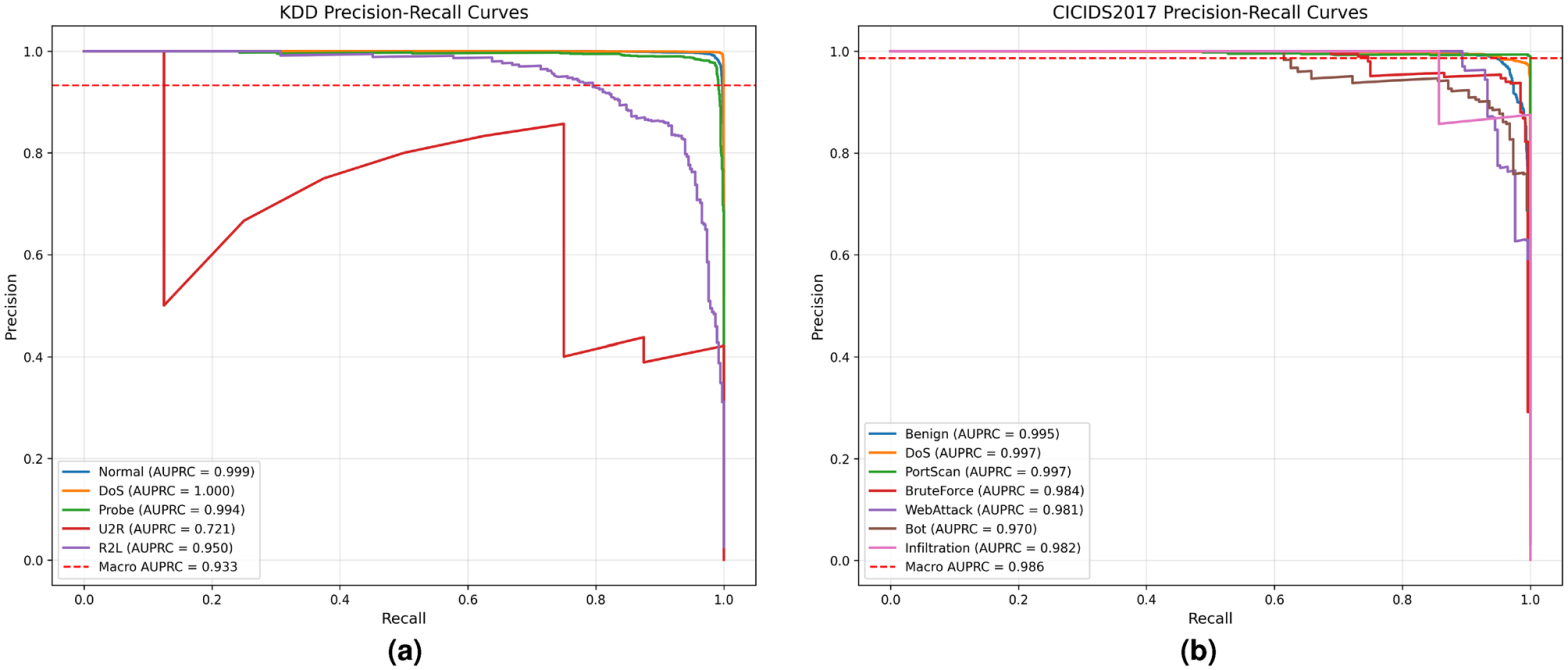
Precision-recall curves.

The Precision-Recall (PR) curve of the NSL-KDD dataset (Fig. 9a) further quantifies this performance: the AUPRC (Area Under the PR Curve) for Normal, DoS, Probe, and R2L traffic reaches 1.000, 1.000, 0.998, and 0.985, respectively, demonstrating near-perfect classification ability.

Notably, even for the U2R attacks with relatively few samples, the model still achieves an AUPRC of 0.761, and the macro-averaged AUPRC reaches 0.949. This result reflects the model’s robustness to class imbalance from the trade-off perspective between precision and recall, aligning with the observation in Fig. 6a.

Similarly, based on the experimental results on CICIDS2017 as presented in Figs. 7b and 9b, we observe that the model can effectively detect malicious traffic even when only a few infiltration attack samples are available. The PR curve of CICIDS2017 provides intuitive support: although Infiltration attack samples are scarce, its AUPRC reaches 1.000. Meanwhile, categories such as Benign, DoS, PortScan, and BruteForce all achieve an AUPRC of 0.998 or higher, with Bot and WebAttack achieving 0.987 and 0.978, respectively, and the macro-averaged AUPRC is 0.994. This consistency between PR curve analysis and confusion matrix results validates the model’s capability to handle rare attack scenarios.

This excellent performance stems from our innovative model architecture:

1. The proposed CCANet effectively extracts spatiotemporal features of traffic data through the combination of CNN and Attention. Specifically, we stack three CCA Blocks, utilizing 1D CNN to capture local spatiotemporal patterns of traffic flow, and employ Attention mechanisms to model global contextual dependencies within the data.
2. The adopted MEMAE (Memory-Augmented Autoencoder) addresses feature limitation issues by amplifying core distinctions between traffic categories through its memory module’s addressing mechanism, resulting in significantly distinct latent features for different classes.
3. Under our multi-task learning framework, auxiliary tasks impose constraints on the main task, compelling the model to learn meaningful features while enhancing generalization capabilities. Furthermore, the implemented Multi-Dilated Local Attention dynamically integrates spatiotemporal and latent features through a dilated attention mechanism, increasing the informativeness and discriminative power of fused features. This architecture provides optimal inputs for subsequent classifiers to distinguish imbalanced classes.

#### Multi-task weight sensitivity analysis

To analyze the sensitivity of hyper parameters $\alpha$ (weight for the main task) and $\beta$ (weight for the auxiliary task) in the Eq. (25).we interpret the experimental results across the NSL-KDD and CICIDS2017 datasets, focusing on ACC, REC, and FPR.

The multi-task weights $\alpha$ and $\beta$ were tuned via grid search on a held-out validation set (10% of the original data). We evaluated $\alpha \in$ 0.6, 0.8, 0.9, 1.1, 1.2 and $\beta \in$ 1.5, 1.3, 1.2, 1.0, 0.9, selecting the combination ($\alpha$=0.9, $\beta$=1.2) that maximized validation accuracy while maintaining a false positive rate below 0.2%. The final model was trained on the combined training and validation sets and evaluated strictly on the untouched test set.

As shown in the Tables 3 and 4 , the results demonstrate that the optimal balance occurs at $\alpha$=0.9, $\beta$=1.2, achieving 99.48% ACC with 0.16% FPR on NSL-KDD and 97.07% REC on CICIDS2017. This confirms the synergy between spatiotemporal features (CNN-Attention) and latent representations (MEMAE).

Imbalanced parameters severely degrade performance. Excessive $\alpha$ suppresses latent feature extraction, reducing REC and increasing FPR, while excessive $\beta$ weakens CNN-Attention’s discriminative capability, causing ACC degradation. Notably, CICIDS2017 exhibits higher sensitivity: increasing $\beta$ from 0.9 to 1.2 reduces REC by 3.0%.

In conclusion, parameter ranges should adapt to dataset complexity. For NSL-KDD with simpler patterns, $\alpha$=0.9 and $\beta$=1.2 remain optimal. In contrast, for CICIDS2017’s complex traffic, $\alpha$=0.9 and $\beta$=1.2 are recommended to balance spatiotemporal pattern capture (main task) and feature robustness (auxiliary task), thereby minimizing FPR.

**Table 3:** The impact of $\alpha$ and $\beta$ on experimental results in the NSL-KDD test dataset.

$\alpha$	$\beta$	ACC%	REC%	FPR%
0.6	1.5	98.78	93.15	0.2
0.8	1.3	99.42	93.21	0.17
**0.9**	**1.2**	**99.48**	**93.57**	**0.16**
1.1	1.0	99.43	93.32	0.19
1.2	0.9	99.39	93.35	0.19

**Table 4:** The impact of $\alpha$ and $\beta$ on experimental results in the CICIDS2017 test dataset.

$\alpha$	$\beta$	ACC%	REC%	FPR%
0.6	1.5	98.43	95.95	0.29
0.8	1.3	98.75	94.23	0.23
**0.9**	**1.2**	**99.08**	**97.07**	**0.17**
1.1	1.0	98.68	96.21	0.24
1.2	0.9	98.64	94.09	0.25

#### Two-factor sweep of memory size and key dimension

Since the CICIDS2017 dataset has more classes compared with the KDD dataset, we choose to conduct the two-factor experiment on this dataset. With parameter combinations as the dimension, this design facilitates the rapid identification of the optimal parameters. Class-wise results are presented in the Table 5. We found that the model achieves the best performance when the Memory Size (N) is 128 and the key dimension (S) is 16.

**Table 5:** Performance of MEMAE on CICIDS2017 dataset under different combinations of memory size (N) and key dimension (S).

Memory size (N)	Key dimension (S)	8	16	32	64
32	F1 score	96.16	94.93	93.86	92.30
	Accuracy	98.02	97.68	98.74	95.64
64	F1 score	92.93	96.87	97.49	97.75
	Accuracy	96.34	98.76	98.97	98.44
128	F1 score	94.20	**96.48**	93.35	95.25
	Accuracy	96.25	**99.08**	97.74	98.55

### Comparisons with other methods

**Table 6:** Model results and comparison on the NSL-KDD dataset.

Model	ACC%	REC%	FPR%
SVM	69.52	–	–
HAST-IDS	93.27	95.85	–
LuNet	99.14	99.02	0.61
Pelican	99.21	99.13	0.65
CNN-BiLSTM	99.22	98.88	0.43
MEMCAIN (ours)	**99.48**	93.57	**0.16**

**Table 7:** Model results and comparison on the CICIDS2017 dataset.

Model	ACC%	REC%	FPR%
LR	87.00	87.00	–
DT	94.00	94.00	–
RF	94.40	94.40	–
CSE-IDS	92.00	–	–
DNN	95.60	95.60	–
MEMCAIN (ours)	**99.08**	**97.07**	0.17

To evaluate the superiority of our model,we compare the performance of MEMCAIN to other state-of-the-art models.

For the NSL-KDD dataset, we compared MEMCAIN with the traditional machine learning model SVM and four deep learning models: HAST-IDS, LuNet^7^ , Pelican^8^, and CNN-BiLSTM^9^. For the CICIDS2017 dataset, we compared MEMCAIN with two deep learning methods, CSE-IDS^10^ and DNN, as well as three machine learning methods: LR, DT, and RF. All models were trained using the same publicly available datasets.

Tables 6 and 7 summarize the multi-class classification performance of our proposed model in comparison with the baseline models.

The experimental results indicate that our model demonstrates competitive advantages relative to baseline methods on specific metrics within our experimental configuration. On the NSL-KDD dataset, compared with the best-performing baseline approach (CNN-BiLSTM), our solution exhibits marginal but consistent improvements, achieving a 0.26% increase in ACC and reduces FPR by 0.27% . When evaluated on the CICIDS2017 benchmark, the proposed architecture shows superior performance over conventional machine learning models in all measured metrics. Compared with the top-performing deep learning baseline (DNN), it achieves a 3.48% improvement in ACC.

### Ablation study

In this section, we conduct an ablation study to validate the effectiveness of MEMCAIN. We compare the complete MEMCAIN with its three variants and two optimal methods, described as follows:

- CCANet: Utilizes only CCANet for model training.
- CCANet+MDA: Connects Multi-Dilated Local Attention (MDA) between CCANet and the classifier.
- CCANet+MEMAE: Integrates CCANet and MEMAE while removing MDA from the feature fusion module.
- CCANet+MEMAE+MDA (MEMCAIN): Integrates CCANet, MEMAE, and MDA.

**Table 8:** Results of the ablation study.

Model	NSL-KDD			CICIDS2017		
	ACC%	REC%	FPR%	ACC%	REC%	FPR%
CCANet	98.60	76.96	0.19	92.59	72.46	1.61
CCANet + MDA	98.87	76.76	0.26	95.41	80.47	0.93
CCANet + MEMAE	98.72	89.16	0.17	95.55	88.76	0.86
CCANet + MEMAE + MDA (MEMCAIN)	99.48	93.57	0.16	99.08	97.07	0.17

Table 8 presents the results of MEMCAIN and its three variants in terms of ACC, REC, and FPR. Using CCANet as the baseline, we observe that when evaluated by REC, both MDA and MEMAE significantly enhance the performance of CCANet on the NSL-KDD and CICIDS2017 datasets. Clearly, integrating these two modules with our proposed CCANet results in a substantial improvement in ACC, REC, and FPR for the full MEMCAIN, especially in REC, on both NSL-KDD and CICIDS2017. This improvement is particularly notable in REC. For instance, on NSL-KDD, REC increases by 16.61%, ACC improves by 0.88%, and FPR decreases by 0.03%. On CICIDS2017, REC rises by 24.61%, ACC improves by 6.49%, while FPR drops by 1.44%.

Through the ablation study, we also observe that in the NSL-KDD dataset, although incorporating the MDA module improves the ACC, it exerts a certain negative impact on the FPR, a phenomenon not seen in CICIDS2017. As illustrated in Figs. 9a and 8a, on the KDD dataset, integrating MDA increases the Macro AUPRC (Area Under the Precision-Recall Curve) from 0.933 to 0.949. Notably, it enhances the performance of hard-to-detect categories such as U2R, with its AUPRC rising from 0.721 to 0.761, which demonstrates the effectiveness of MDA in extracting key local features. However, the PR curve of the U2R category exhibits significant fluctuations, indicating that the model still struggles to distinguish U2R from normal traffic—this aligns with the characteristic that U2R attacks share similar local features with normal traffic, and we attribute this to the local attention bias of MDA. In contrast, as shown in Figs. 8b and 9b, on the CICIDS2017 dataset, adding the MDA module increases the Macro AUPRC from 0.986 to 0.994, and all categories show remarkable performance improvements. For instance, Bot attacks have distinct local feature differences from normal traffic, allowing the advantages of MDA to be fully leveraged. Therefore, to alleviate the local attention bias of MDA when handling traffic with high feature similarity, we introduce MEMAE as an auxiliary task, utilizing the global consistent features provided by the memory module for effective supplementation.

The compressed intermediate features of MEMAE complement global structural information, reducing misclassification due to local similarities and significantly decreasing FPR. As a result, the full MEMCAIN, incorporating MDA and MEMAE, utilizes MEMAE’s global semantic priors to adjust MDA’s attention distribution. This process enhances class separation in the feature space, ultimately improving classification performance.

## Conclusion and future work

This study proposes MEMCAIN, a multi-task feature fusion framework for network intrusion detection, pioneering the integration of a memory-enhanced autoencoder as an auxiliary task to enhance discriminative capability in anomaly traffic identification. The key contributions and findings are summarized as follows:

1. Spatiotemporal feature learning via CCA Block We design the CCA Block by synergizing attention mechanisms with 1D convolutional networks and contrast normalization layers. Cascaded deployment of CCA blocks enables efficient spatiotemporal feature extraction from raw network traffic, significantly improving scalability and adaptability for large-scale network intrusion detection systems (NIDS).
2. Multi-task synergism through dual-task architecture MEMCAIN combines CCANet with a memory-enhanced autoencoder (auxiliary task) to achieve complementary feature fusion. The memory module mitigates class imbalance through latent traffic prototype learning while enhancing feature discriminability. Joint end-to-end optimization ensures mutual reinforcement between tasks: CCANet refines anomaly detection precision, whereas the memory autoencoder enforces global discriminative constraints via prototype-guided regularization.
3. Empirical superiority validation Ablation studies and comparative experiments on NSL-KDD and CICIDS2017 demonstrate MEMCAIN’s superiority over state-of-the-art methods in accuracy (ACC) and false positive rate (FPR). The integration of MDA (Multi-Dilated Local Attention) and MEMAE (Memory-Enhanced Autoencoder) achieves ACC improvements of 0.24% on NSL-KDD and 3.48% on CICIDS2017 compared to optimal baselines, alongside FPR reductions up to 0.27%. These findings substantiate the critical role of multi-task feature fusion and memory-driven prototype learning in advancing network anomaly detection.

### Future directions

1. CCA Block optimization potential Ablation results reveal that CCANet exhibits suboptimal discriminative power in distinguishing feature-wise similarities (e.g. between U2R and R2L attacks in NSL-KDD). Future work will focus on architectural refinements to enhance its separability.
2. MDA’s pseudo-similarity challenge While MDA improves feature extraction, its local window similarity computation struggles to differentiate legitimate intra-class variations from cross-category pseudo-similarities. Though MEMAE partially addresses this through supplementary feature constraints, subsequent research will explore contrastive learning or adversarial separation mechanisms to refine MDA.

## Data Availability

The datasets analysed during the current study are available in NSL-KDD and CICIDS-2017. NSL-KDD: https://www.unb.ca/cic/datasets/nsl.html CICIDS-2017: https://www.unb.ca/cic/datasets/ids-2017.html

## References

[1] He, K., Kim, D. D. & Asghar, M. R. Adversarial machine learning for network intrusion detection systems: A comprehensive survey. *IEEE Commun. Surv. Tutor.* **25**, 538–566 (2023).

[2] Qu, H., Qiu, Z., Tang, X., Xiang, M. & Wang, P. Incorporating unsupervised learning into intrusion detection for wireless sensor networks with structural co-evolvability. *Appl. Soft Comput.* **71**, 939–951 (2018).

[3] Lin, K., Xu, X. & Xiao, F. Mffusion: A multi-level features fusion model for malicious traffic detection based on deep learning. *Comput. Netw.* **202**, 108658 (2022).

[4] Ye, Z. & Yu, J. Multi-level features fusion network-based feature learning for machinery fault diagnosis. *Appl. Soft Comput.* **122**, 108900 (2022).

[5] Ahmad, M. B., Iqbal, M. J. & Rahim, A. Performance comparison of support vector machine, random forest, and extreme learning machine for intrusion detection. *IEEE Access* **6**, 33789–33795 (2018).

[6] Wang, W. et al. HAST-IDS: Learning hierarchical spatial-temporal features using deep neural networks to improve intrusion detection. *IEEE Access* **6**, 1792–1806 (2017).

[7] Wu, P. & Guo, H. LuNET: A deep neural network for network intrusion detection. In *IEEE Symposium Series on Computational Intelligence (SSCI)*, 617–624 (IEEE, 2019).

[8] Wu, P., Guo, H. & Moustafa, N. Pelican: A deep residual network for network intrusion detection. In *2020 50th Annual IEEE/IFIP International Conference on Dependable Systems and Networks Workshops (DSN-W)*, 55–62 (IEEE, 2020).

[9] Sinha, J. & Manollas, M. Efficient deep CNN-BiLSTM model for network intrusion detection. In *Proceedings of the 2020 3rd International Conference on Artificial Intelligence and Pattern Recognition*, 223–231 (2020).

[10] Gupta, N., Jindal, V. & Bedi, P. CSE-IDS: Using cost-sensitive deep learning and ensemble algorithms to handle class imbalance in network-based intrusion detection systems. *Comput. Secur.* **112**, 102499 (2022).

[11] Vinayakumar, R. et al. Deep learning approach for intelligent intrusion detection system. *IEEE Access* **7**, 41525–41550 (2019).

[12] Lan, J. et al. MEMBER: A multi-task learning model with hybrid deep features for network intrusion detection. *Comput. Secur.* **123**, 102919 (2022).

[13] Liu, Q., Wang, D., Jia, Y., Luo, S. & Wang, C. A multi-task based deep learning approach for intrusion detection. *Knowl.-Based Syst.* **238**, 107852 (2022).

[14] Nie, F., Liu, W., Liu, G. & Gao, B. M2VT-IDS: A multi-task multi-view learning architecture for designing IoT intrusion detection system. *Internet Things* **25**, 101102 (2024).

[15] Telikani, A. et al. A cost-sensitive machine learning model with multitask learning for intrusion detection in IoT. *IEEE Trans. Ind. Inf.* **20**, 3880–3890 (2023).

[16] Eid, A. M., Soudan, B., Nassif, A. B. & Injadat, M. Enhancing intrusion detection in IIoT: Optimized CNN model with multi-class SMOTE balancing. *Neural Comput. Appl.* **36**, 14643–14659 (2024).

[17] Andresini, G., Appice, A., De Rose, L. & Malerba, D. GAN augmentation to deal with imbalance in imaging-based intrusion detection. *Futur. Gener. Comput. Syst.* **123**, 108–127 (2021).

[18] Ding, H., Sun, Y., Huang, N., Shen, Z. & Cui, X. TMG-GAN: Generative adversarial networks-based imbalanced learning for network intrusion detection. *IEEE Trans. Inf. Forensics Secur.* **19**, 1156–1167 (2023).

[19] Ren, K., Yuan, S., Zhang, C., Shi, Y. & Huang, Z. CANET: A hierarchical CNN-attention model for network intrusion detection. *Comput. Commun.* **205**, 170–181 (2023).

[20] Gong, D. et al. memorizing normality to detect anomaly: memory-augmented deep autoencoder for unsupervised anomaly detection. In *Proceedings of the IEEE/CVF International Conference on Computer Vision*, 1705–1714 (2019).

